# Supplementary material for: Generation of an expandable intermediate mesoderm restricted progenitor cell line from human pluripotent stem cells
Source: eLife. 2015 Nov 10;4:e08413. doi: 10.7554/eLife.08413 (PMC4631902; doi:10.7554/eLife.08413)
Supplement: Supplementary file 1. — This table provides the complete list of genes with similar expression levels in MP cells and mesoderm (MP + ME, panel A), and of genes expressed only in MP cells (MP only, panel B). DOI: http://dx.doi.org/10.7554/eLife.08413.026 [file elife08413s001.pdf]

Supplementary file 1A: 244 MP and ME genes

AATF	CCT8	ERLIN1	LIPG	PC	SAR1A	TNFAIP8L2-
ABCD3	CD44	EXOSC4	LOC440925	PCDHGC4	SDCCAG3	SCNM1
ABCF3	CENPB	FAIM	LPAR6	PDE3B	SFXN2	TNFRSF11B
ABCG2	CLPB	FAM13A	LRFN5	PGGT1B	SKA1	TOE1
ACAT1	CMKLR1	FAM181B	LRRC55	PHAX	SLC25A12	TOMM34
ADAMTS15	CNPY2	FAM24B	LRRTM1	PHLDA2	SLC26A2	TOPBP1
AGPAT5	CNPY3	FDX1L	LSG1	PIP4K2B	SLC35E1	TOR1B
ALDH1A2	CNST	FEN1	MAD2L1	PITHD1	SLC35G1	TPRKB
ALG2	COMMD9	FRMD8	MAD2L1BP	PNO1	SLIT2	TRAF2
AP1AR	CRABP2	GABBR1	MAFA	POLR2B	SNAI1	TRMU
APEX1	CWF19L1	GLMN	MAP2K2	POLR2D	SNAI2	TRPM7
APLNR	CYBASC3	GLRX3	MCM8	POLR2J	SOD1	TRUB2
ATOH8	DCBLD1	GOSR2	MCOLN3	PRADC1	SP5	UBE2L3
ATP5F1	DDI2	GRINL1A	MDFIC	PRKAG1	SP6	UFD1L
BAG1	DDX19A	GRPEL1	MESP2	PRPF4	SPATA5L1	UNC5C
BAZ1A	DENR	GTF2H1	MINPP1	PRPF6	SPC25	USP33
BGN	DGCR2	GTF3A	MKI67IP	PSMD6	SSBP1	USP5
BTBD1	DHX34	GYPC	MPHOSPH10	PSMD7	STOML2	UTP18
BZW1	DIS3	HAT1	MSX1	PTDSS2	STRAP	WDR55
C14orf169	DIS3L	HMGB1	MTA2	QTRTD1	SUB1	WDR81
C16orf53	DKK1	HMX1	MTRR	RAB35	TACR1	WNT5A
C1orf151	DLL3	HNRNPA1	NBN	RARS	TAF12	YBX1
C1orf174	DNAJA1	HOXB2	NCBP2	RASL10B	TAF9	ZBTB2
C1orf52	DNAJC25	IRX2	NDST1	RASSF9	TAOK2	ZDHHC6
C3orf14	DNAJC7	IRX5	NDUFS1	RBP1	TCEB3	ZFP64
C5orf43	DONSON	ISCA1	NHEJ1	RCC1	TDG	ZMPSTE24
C6orf204	DRG2	ISLR	NOL8	RFC2	TENC1	ZNF100
C7orf16	EEF1B2	ISY1	NTS	RHOBTB2	TFAM	ZNF17
CAND1	EGFLAM	JOSD2	NUBP1	RHOQ	THEM4	ZNF200
CANX	EID1	KAZN	NUP50	RNF20	THOC1	ZNF286A
CAPRIN2	EIF1AD	KCNK17	OBFC2B	RPSA	THOC5	ZNF347
CCDC99	EIF2B1	KHDRBS3	OLFM1	RRAGA	TIMM17A	ZNF45
CCNE2	EMILIN1	KIAA0226L	OLFML3	SAC3D1	TM2D2	ZNF511
CCNF	EMP2	KIAA1429	PANK1	SALL1	TMEM70	ZNF639
CCT6A	ENOX2	LEF1	PANX1	SAMD1	TNFAIP2	ZNF668

Supplementary file 1B: 140 MP genes

ACTA1	C12orf23	DYNC1I1	HHIPL2	LRRN4	PGM3	SVEP1
ACTC1	C12orf35	ECE1	HS3ST3A1	MAB21L2	PGM5	TANC1
ADA	C6orf138	EMILIN2	HSDL2	MAGEB3	PHLDA3	TFPI
ADAM12	C9orf21	FAM123C	HSPA1B	MALT1	PLCB1	TMED7
ADAMTS12	CALU	FAM78A	HTRA1	MMP2	PLEC	TMEM107
AEBP1	CBLN2	FBN2	IFI16	MOGS	PMP22	TMEM185A
ALPK2	CCNB1IP1	FMOD	IL11	MPST	PPP1R2	TMF1
AMN1	CHIC2	FN1	IL6	MSX2	PSTPIP2	TNFAIP3
AMOTL1	CHST11	FOXC2	ISL1	MYL4	PXDN	TNFRSF19
AP3S1	CLSTN2	FSHR	ITGA8	MYLK3	RASGRP1	TNNT1
ARSK	CNOT2	GNG11	ITGA9	NFATC1	RASGRP3	TPK1
ART5	COL1A2	GOLIM4	JPH2	NKX3-1	RGS4	TTC9C
ATP8B3	CREG1	GRAP2	KDELC1	NPPA	RIPPLY1	TWIST1
B2M	CRELD2	GREM1	KIAA1462	NRP1	SCGB1A1	VPS29
BAMBI	CTNNB1	GSN	KIF26B	PAPPA	SCYL1	VSTM2L
BIN1	DDB1	GYPB	KLK6	PCSK2	SEMA3G	WDR20
BMP4	DGKI	GYPE	KLK7	PCYOX1	SESN1	YIPF5
BMP5	DNAJC14	HAPLN1	LAMC1	PDGFRA	SFRP5	ZAP70
BMPER	DOK4	HAS2-AS1	LNPEP	PDGFRB	SPSB1	ZNF611
BSG	DVL1	HCN1	LRRC32	PGF	SRP54	ZNF702P
